# Bovine Respiratory Syncytial Virus Genome Sequences from Cattle with Clinical Respiratory Disease in Kansas, 2021

**DOI:** 10.1128/mra.00313-22

**Published:** 2022-04-12

**Authors:** Andrea Lu, Steven Stancic, Franco Matias-Ferreyra, Gregg Hanzlicek, Rachel Palinski

**Affiliations:** a Kansas State Veterinary Diagnostic Laboratory, Manhattan, Kansas, USA; Queens College CUNY

## Abstract

We report two near-complete bovine respiratory syncytial virus genome sequences collected from 10-month-old cattle with respiratory disease in Kansas in December 2021. No other respiratory pathogens were confirmed in the samples. These genome sequences update the currently circulating BRSV field strains in the United States.

## ANNOUNCEMENT

Bovine respiratory syncytial virus (BRSV; genus *Pneumovirus*, family *Paramyxoviridae*) is a negative-sense, single-stranded RNA virus that contributes to bovine respiratory disease (BRD) in U.S. cattle (1). Symptoms associated with BRSV range from mild to severe and are generally characterized by fever, coughing, and nasal discharge (2). Multiple vaccines are available to prevent BRSV infection; however, the high mutation rate allows the virus to escape a vaccinated immune system (3, 4). As severe BRD cases with BRSV are rare, little emphasis has been placed on BRSV surveillance.

Lungs from two concomitant cases of BRD followed by mortality were submitted to the Kansas State Veterinary Diagnostic Laboratory (KSVDL) for testing. The samples were collected from 10-month-old vaccinated cattle on the same farm in Kansas, USA.

At KSVDL, RNA was extracted using the Direct-zol RNA kit (Zymo Research), followed by first-strand cDNA synthesis with published primers (Superscript III, Invitrogen) and second-strand synthesis with a DNA polymerase and published primers (TaKaRa Bio) (5–7). The primers were removed (HighPrep PCR cleanup system; MagBio), and libraries were prepared using the Nextera XT v2 dual-indexing kit (Illumina). The libraries were run on the Illumina MiSeq platform using a 300-cycle v2 cartridge. All sample preparation steps were performed following the manufacturer’s protocols.

The raw reads were trimmed and *de novo* assembled, and the closest reference sequence was determined using BLASTn (Table 1). This sequence, BRSV/USII/S1 (GenBank accession number KU159366), was collected in 2015 from cattle with respiratory disease (8). The trimmed reads were aligned to the reference sequence to extract the most complete genome and consensus sequences, resulting in average coverages of 1,301× and 10,139× across the BRSV/KS/467/2021 and BRSV/KS/090/2021 genomes, respectively. All bioinformatics steps were performed using default parameters in CLC Genomics Workbench v21.0.4.

**TABLE 1:** Assembly statistics and overview of nucleotide changes in BRSV genomes

Characteristic	Length (nt)	Data for strain:^*a*^		Difference (nt) between EO
		BRSV/KS/467/2021	BRSV/KS/090/2021	
Gene				
5′ UTR^*b*^	88	5	0	5
NS1	411	4	40	36
NS2	375	7	57	59
N	1,176	6	4	4
P	726	1	6	7
M	771	8	7	3
SH	246	2	1	1
G	792	16	19	15
F	1,725	12	19	15
M2	826	11	17	12
L	6,489	40	139	123
3′ UTR	235	4	0	4
Total	14,440	107	309	275
Assembly statistics				
No. of trimmed reads		1,923,701	1,430,832	
Avg read length (nt)		141	138	
No. of contigs		124	43	

a Difference (nt) from the closest GenBank reference sequence, BRSV/USII/S1 (GenBank accession no. KU159366), or between each other (EO).

b UTR, untranslated region.

The two near-complete genomes are 15,122 and 14,801 nucleotides (nt) long, with a GC content of 33.4% and 10 open reading frames encoding proteins of the expected sizes (Table 1) (1). The genomes are 97.7% identical to each other and 99.1 or 97.5% identical to the BRSV/USII/S1 reference sequence. The individual genes in the two genomes were 87.6 to 99.6% identical to each other and 88.1 to 99.6% identical to those in BRSV/USII/S1, corresponding to differences of 1 to 59 nt. All nucleotide differences are listed in Table 1. Other pathogens detected in the sample included Mycoplasma bovis and influenza D virus.

Deep sequencing has allowed for the rapid identification and genotyping of common and uncommon outbreak pathogens. This presents a unique way to implement a timely response.

### Data availability.

The consensus genomes were deposited at GenBank under accession numbers OM328114 to OM328115 and in the Sequence Read Archive under accession numbers SAMN26796202 and SAMN26796201. This project references the first version of the sequences.
